# FoxO1 as a tissue-specific therapeutic target for type 2 diabetes

**DOI:** 10.3389/fendo.2023.1286838

**Published:** 2023-10-23

**Authors:** Nicole A. Teaney, Nicole E. Cyr

**Affiliations:** ^1^ Stonehill College, Neuroscience Program, Easton, MA, United States; ^2^ Stonehill College, Department of Biology, Easton, MA, United States

**Keywords:** FoxO1, type 2 diabetes, insulin resistance, Akt, metabolism

## Abstract

Forkhead box O (FoxO) proteins are transcription factors that mediate many aspects of physiology and thus have been targeted as therapeutics for several diseases including metabolic disorders such as type 2 diabetes mellitus (T2D). The role of FoxO1 in metabolism has been well studied, but recently FoxO1’s potential for diabetes prevention and therapy has been debated. For example, studies have shown that increased FoxO1 activity in certain tissue types contributes to T2D pathology, symptoms, and comorbidities, yet in other tissue types elevated FoxO1 has been reported to alleviate symptoms associated with diabetes. Furthermore, studies have reported opposite effects of active FoxO1 in the same tissue type. For example, in the liver, FoxO1 contributes to T2D by increasing hepatic glucose production. However, FoxO1 has been shown to either increase or decrease hepatic lipogenesis as well as adipogenesis in white adipose tissue. In skeletal muscle, FoxO1 reduces glucose uptake and oxidation, promotes lipid uptake and oxidation, and increases muscle atrophy. While many studies show that FoxO1 lowers pancreatic insulin production and secretion, others show the opposite, especially in response to oxidative stress and inflammation. Elevated FoxO1 in the hypothalamus increases the risk of developing T2D. However, increased FoxO1 may mitigate Alzheimer’s disease, a neurodegenerative disease strongly associated with T2D. Conversely, accumulating evidence implicates increased FoxO1 with Parkinson’s disease pathogenesis. Here we review FoxO1’s actions in T2D conditions in metabolic tissues that abundantly express FoxO1 and highlight some of the current studies targeting FoxO1 for T2D treatment.

## 1 Introduction

T2D is a global health concern. A recent study reported that T2D accounts for more than 90% of the 529 million diabetes cases in 2021 (1). Moreover, the incidence of T2D is expected to increase in adults and children over the next thirty years. T2D is characterized by insulin resistance and hyperglycemia. Consequently, its treatments target these metabolic states. FoxO1 is a transcription factor that mediates many cellular functions including inflammation, oxidative stress (OS), cell proliferation, apoptosis, and autophagy which contribute to the regulation of insulin signaling and glycemia. However, whether increased FoxO1 transcriptional activity exacerbates or alleviates T2D appears to be tissue-type specific and may depend on cellular conditions (2, 3). Recent studies have called attention to these FoxO1 controversies (4, 5). The current review describes FoxO1’s actions in T2D conditions in metabolic tissues that highly express FoxO1 and highlights some of the current progress in this field.

## 2 FoxO1 structure, function, and regulation

**Figure 1:**
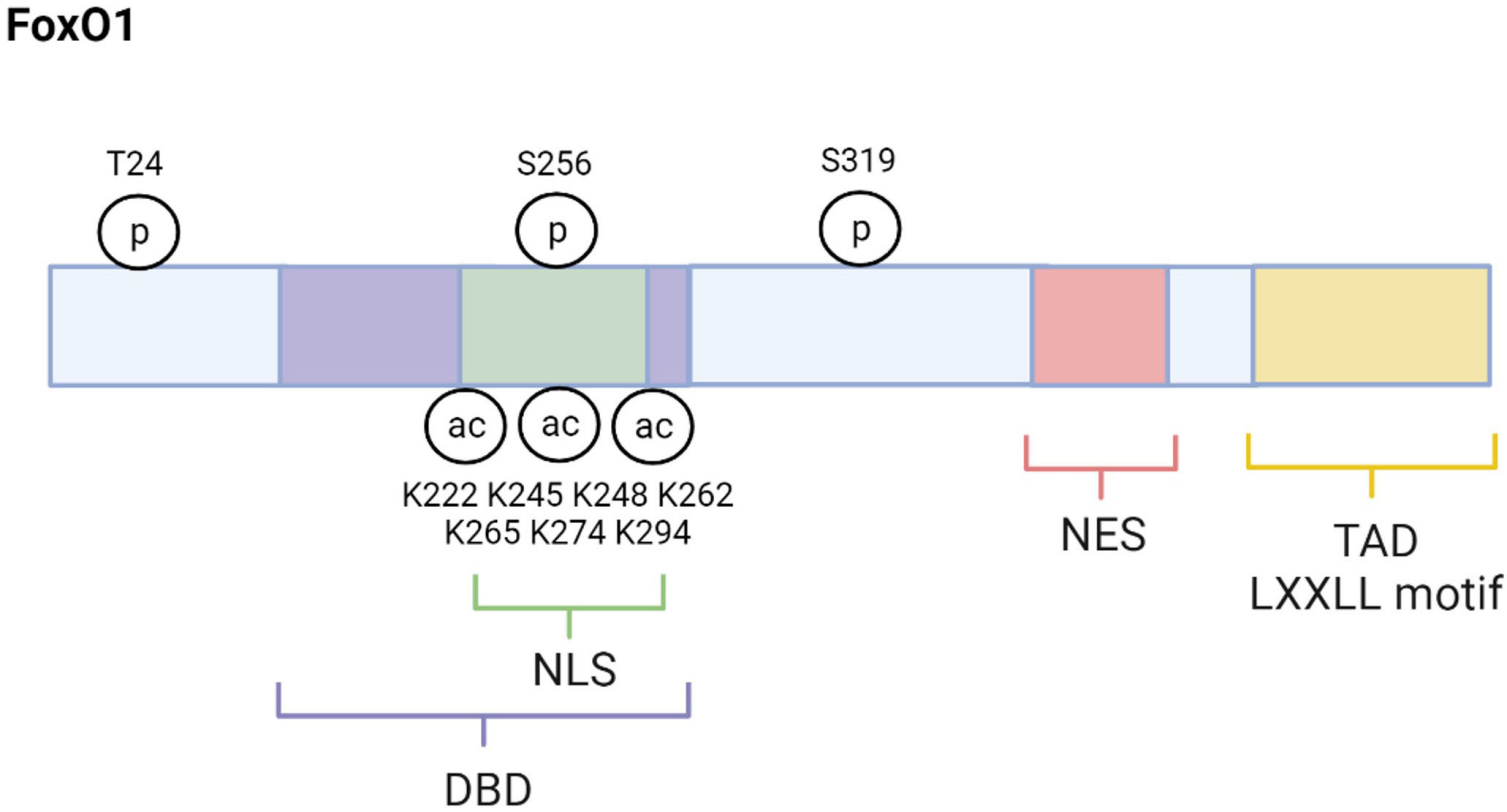
FoxO1 structure and regulatory sites. FoxO1 structure is composed of several functional domains, including DNA-binding domain (DBD), nuclear location signal (NLS), nuclear export signal (NES), and transactivation domain (TAD). The major regulatory sites discussed in this review are also shown. Created with BioRender.com.

FoxO1 (also forkhead in rhabdomyosarcomam (FKHR)) is one of four mammalian isoforms belonging to the FoxO transcription factor family. The others include FoxO3, FoxO4, and FoxO6 (5–7). These FoxO proteins are mainly localized in the nucleus and exert their actions through their ability to regulate the transcription of specific genes. However, they can also be found in the cytoplasm where they are considered inactive. Although their primary action occurs in the nucleus, new evidence indicates that FoxO proteins can be localized to the mitochondria where they have varying effects on mitochondrial function (8–10). FoxO1 structure, as depicted in **Figure 1**, is similar to the other mammalian FoxO isoforms. For example, FoxO1, FoxO3, FoxO4, and FoxO6 each contain a highly conserved winged‐helix or forkhead DNA‐binding domain (DBD) and bind to the consensus DNA sequence 5′‐TTGTTTAC‐3′ (11, 12). Their remaining functional domains consist of a nuclear localization signal (NLS), a nuclear export signal (NES), and a transactivation domain (TAD). FoxO amino acid composition varies slightly among species. FoxO1 consists of about 655 amino acids, FoxO3 has about 670 amino acids, Foxo4 has about 505 amino acids, and FoxO6 has about 490 amino acids with the greatest amino acid differences among these proteins occurring in the TAD (6). FoxO1, FoxO3 and FoxO4 are ubiquitously expressed (13–17). For example, FoxO1 is predominantly expressed in adipose tissue but also abundantly found in other metabolic tissues such as liver, skeletal muscle, pancreas, and brain. FoxO3 is primarily expressed in liver and FoxO4 is largely expressed in muscle. In contrast, expression of FoxO6 is mostly concentrated in the brain (18).

FoxO1, FoxO3, FoxO4, and FoxO6 control some of the same cellular functions by regulating genes involved in the cell cycle, OS, inflammation, glucose metabolism, and autophagy (6, 19). However, whole-body knockout studies in mice reveal a unique role in specific cellular functions for each FoxO isoform. For example, lack of FoxO1 is lethal on embryonic day 10.5 due to vasculature defects indicating an important role for FoxO1 in angiogenesis during development (20). FoxO3 has been shown to regulate reproduction as FoxO3 null female mice exhibit impaired ovarian follicular development and infertility. While one study reported no phenotypic differences between FoxO4 null mice and wild-type mice (20), another showed that FoxO4 knockout mice were at a higher risk of developing colitis when challenged with increased inflammation due to elevated levels of NF-κB, which is a transcription factor that mediates the immune response. These results suggest that FoxO4 regulates gut inflammation and health (21). Salih et al. (22) demonstrated that FoxO6 is a key mediator of memory consolidation and controls the transcription of synaptic proteins in hippocampal neurons involved in long-term memory.

Activity of all FoxOs depends on post-translational modifications. While processes such as ubiquitination, methylation, and glycosylation can alter FoxOs, phosphorylation and acetylation are the principal post-translational modifications regulating FoxO activity (5, 6). Phosphorylation strongly regulates FoxO activity as it affects FoxO’s cellular localization, thus transcriptional capacity. There are several kinases that phosphorylate FoxO proteins and cause these proteins to be shuttled out of the nucleus into the cytoplasm where they are inactive. These FoxO inactivating kinases include AKT (Protein kinase B), extracellular signal- regulated kinase (ERK), and cyclin-dependent kinase (CDK) 1/2 (23–26). In contrast, phosphorylation by the FoxO activating kinases such as adenosine monophosphate-activated (AMPK), Jun- N-terminal kinase (JNK), mammalian sterile 20-like kinase (MST1), and Protein Kinase RNA-Like ER Kinase (PERK) result in nuclear localization and nuclear retention of FoxO proteins which increases their transcriptional activity (27–30).

Many physiological conditions regulate FoxO activating and inactivating kinases including conditions that are altered in the pathogenesis and pathology of T2D. For example, JNK is a FoxO-activating kinase known to increase in response to elevated inflammation and OS, which are conditions that contribute to the development of T2D (28, 31). JNK phosphorylates FoxO4 specifically at T447 and T451 which promotes its nuclear transport and activity (28). Although FoxO1 is also phosphorylated and translocated into the nucleus by JNK the exact mechanism has not yet been described (32). Activation of FoxOs by JNK increases the transcription of certain antioxidants like catalase and manganese superoxide dismutase (MnSOD, or SOD2) to combat OS (28, 31). Therefore, the post-translational phosphorylation of FoxOs by JNK helps to protect against OS and T2D. In contrast to JNK, phosphorylation of FoxO1, FoxO3, and FoxO4 by the active, phosphorylated AKT (pAKT) causes the exportation and sequestration of these FoxO1 proteins into the cytoplasm (33). In particular, AKT phosphorylates FoxO1 at T24, S256, and S319 sites (23) (**Figure 1**). AKT phosphorylation of FoxO3 occurs at T32, S253, and S315 (34, 35). T28, S193, and S258 were identified as putative sites for AKT phosphorylation of FoxO4, but studies indicate that insulin stimulated AKT phosphorylates FoxO4 specifically at S193, and S258 (36, 37). Unlike the other FoxO isoforms, FoxO6 is less sensitive to AKT-mediated inactivation because it contains T26 and S184 as AKT phosphorylation sites (38). In fact, research indicates that AKT suppression of FoxO6 activity is independent of cytoplasmic translocation.

**Figure 2:**
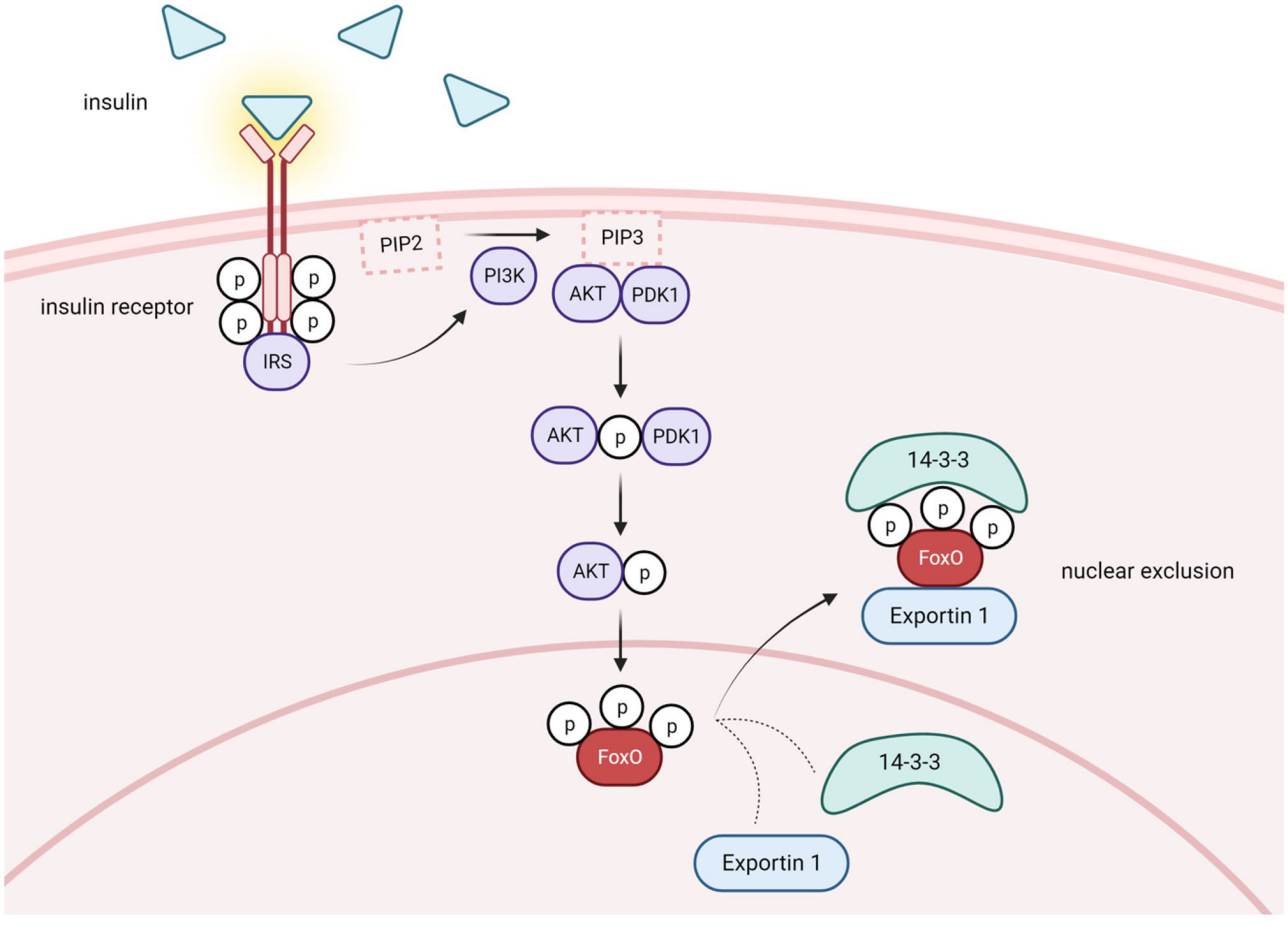
Postprandial conditions lead to FoxO nuclear exclusion via PI3K/AKT pathway activation. Under healthy conditions in the postprandial state, insulin is released from pancreatic B-cells and binds to insulin receptors in several different tissues. Activation of the receptor tyrosine-kinases leads to recruitment of insulin receptor substrate (IRS) and initiation of the PI3K/AKT signaling cascade. PI3K phosphorylates phosphatidylinositol 4,5-bisphosphate (PIP_2_), generating phosphatidylinositol-3,4,5-triphosphate (PIP_3_). Subsequently, PDK1 phosphorylates and activates AKT. Phosphorylated AKT phosphorylates FoxO, which promotes recruitment of Exportin 1 and nuclear exclusion. 14-3-3 protein is also recruited and covers FoxO's NLS, inhibiting nuclear reentry. Activation of the PI3K/AKT pathway causes cytoplasmic retention and decreases the transcriptional capacity of FoxO. Created with BioRender.com.

**Figure 3:**
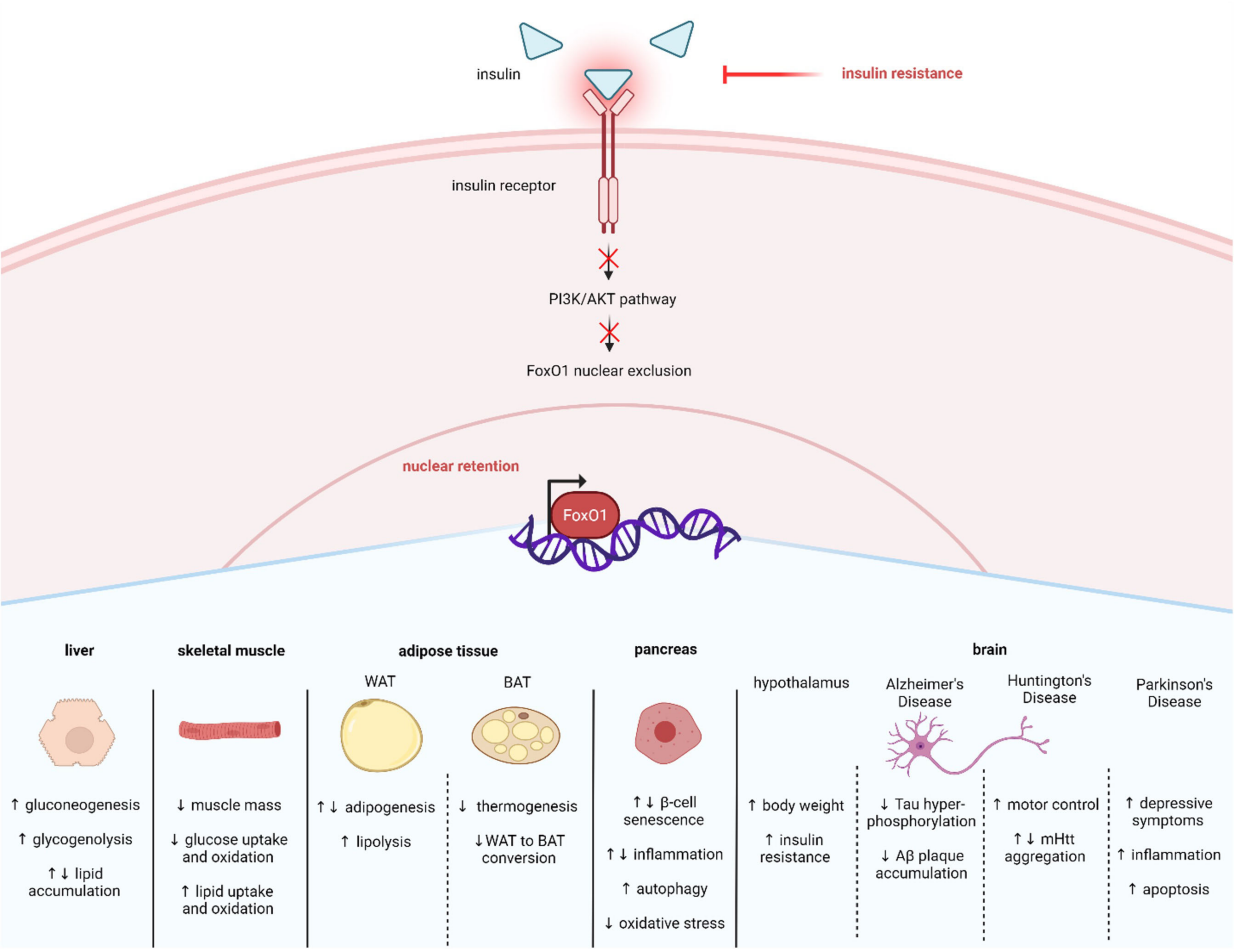
Insulin resistance affects many tissues via FoxO1 nuclear retention. Insulin resistance in T2D results in less activation of the PI3K/AKT pathway. Decreased insulin induced PI3K/AKT signaling enhances FoxO1 nuclear retention and activity, which mediates many cellular responses across tissue types, including liver, skeletal muscle, adipose, pancreas, and brain. Created with BioRender.com.

One cellular condition in which pAKT is enhanced is when insulin levels are high postprandially. Insulin binding to its receptor recruits insulin receptor substrate (IRS), which in turn, activates PI3K (phosphatidylinositol-3 kinase) which activates AKT (**Figure 2**). The phosphorylation of FoxO proteins by pAKT has been well studied and shown to augment 14-3-3 protein binding to phosphorylated serine and threonine residues on pFoxO, which causes a conformational change that exposes a leucine-dense NES and subsequently recruits nuclear export factor Exportin 1 to shuttle FoxOs to the cytoplasm (39–44) (**Figure 2**). Furthermore, 14-3-3 binding covers the FoxO NLS, which inhibits nuclear reentry (45, 46). Therefore, FoxO activity is reduced in the fed state. In contrast, during insulin resistance conditions, insulin’s activation of PI3K/AKT signaling is compromised. Consequently, AKT-mediated FoxO inactivation is reduced and FoxO activity increases in T2D (**Figure 3**).

In addition to phosphorylation, acetylation is an important post-translational modification that affects FoxO activity. Histone acetyltransferases, like CREB-binding protein (CBP) and p300, acetylate FoxOs at the K222, K245, K248, K262, K265, K274 and K294 residues, which decreases DNA binding affinity and inhibits transcriptional activity (47, 48) (**Figure 1**). Furthermore, acetylation of FoxOs increases their sensitivity to inactivation via phosphorylation and nuclear exclusion (47). Interestingly, FoxO inactivation by CBP/p300 is enhanced during OS conditions which can lead to a decrease in FoxO1 upregulation of antioxidants like MnSOD and put the cell at risk for apoptosis (49, 50). However, there are mechanisms to increase FoxO1 activity during OS. For example, JNK activates FoxO proteins even under conditions of low OS (28). Furthermore, the inactivation of FoxOs by CBP/p300 acetylation can be reversed by deacetylating and thus re-activating FoxOs (49). Histone deacetylases like the NAD^+^-dependent Sirtuins, including the mammalian Sirtuin 1 (SIRT1), deacetylate FoxOs (51). Research reveals that SIRT1 deacetylation of FoxOs during OS can reverse the CBP/p300 effects on FoxO inhibition. For example, SIRT1 deacetylation and activation of FOXOs upregulates antioxidants and genes involved in DNA repair to mitigate OS and thus help to prevent the development of OS-related diseases like T2D (49, 52, 53).

SIRT1 also plays a significant role in regulating glucose and lipid metabolism (54, 55). Furthermore, SIRT1 is altered in metabolic conditions like T2D. For example, studies have reported that SIRT1 levels are lower in serum and muscle cells of patients with T2D, suggesting that loss of SIRT1 activity may contribute to T2D pathology (56, 57). Consistent with this idea, other studies have shown that increasing SIRT1 can alleviate T2D symptoms. For example, overexpression of SIRT1 in the liver of diabetic db/db mice improves insulin resistance and glucose tolerance (58). SIRT1’s actions are exerted in part by regulating FoxO1. Specifically, SIRT1 interacts with FoxO1 at its LXXLL motif located at amino acids 459–463 which enables deacetylation to occur (51) (**Figure 1**). One mechanism through which SIRT1 improves T2D symptoms is by deacetylating and thus activating FoxO1 in adipocytes and increasing FoxO1’s interaction with its coactivator CCAAT/enhancer-binding protein α (C/EBPα) to upregulate the transcription of the insulin-sensitizing hormone adiponectin (59). However, SIRT1 can also act to mitigate T2D by enhancing AKT-mediated FoxO1 inactivation in tissues like liver and skeletal muscle (60, 61). Therefore, increased SIRT1 can improve T2D symptoms by either activating or inactivation FoxO1. The current review focuses on FoxO1 regulation in T2D within specific metabolic tissues, including liver, skeletal muscle, adipose tissue, pancreatic β-cells, and brain (**Figure 3**).

## 3 FoxO1 and T2D – liver

FoxO proteins are known to mediate glucose metabolism. In the liver, glucose is largely produced from glycogen, the stored form of carbohydrates, by the process of glycogenolysis. However, glucose can also be synthesized in the liver from other sources such as pyruvate, glycerol, lactate, and amino acids through gluconeogenesis, especially during conditions when glycogen is depleted such as a prolonged fast (62). Activation of FoxOs promotes hepatic glucose production by increasing the expression of glucose 6-phosphatase (G6PC), fructose 1,6-bisphosphatase, and phosphoenolpyruvate carboxykinase (PEPCK) which are enzymes involved in gluconeogenesis (63, 64). The enzyme G6PC catalyzes the conversion of glucose-6-phosphate to glucose in gluconeogenesis as well as in glycogenolysis. Therefore, FoxOs increase hepatic glucose levels via both pathways. FoxO’s control of gluconeogenesis and glycogenolysis is mainly regulated by insulin signaling. For example, in the fed state, insulin levels increase and stimulate the PI3K/AKT pathway, which will export FoxO out of the nucleus and reduce FoxO’s transcriptional activity (**Figure 2**). Thus, FoxO inactivation contributes to insulin’s ability to lower blood glucose levels by decreasing hepatic glucose production and output. In contrast, during conditions when insulin signaling is diminished, such as in fasting and insulin resistance states, AKT-mediated nuclear exclusion of FoxO decreases, which causes an overall increase in FoxO transcriptional activity to elevate hepatic glucose production and output (**Figure 3**). The nutrient sensor SIRT1 has been shown to facilitate AKT-mediated effects on FoxO1 and gluconeogenesis. For example, liver-specific SIRT1 deficiency increases AKT which suppresses FoxO1 causing the downregulation of G6PC and PEPCK which decreases gluconeogenesis and lowers blood glucose levels (60). Liver-specific genetic studies indicate that while FoxO3 may contribute, FoxO1 is the predominant isoform regulating gluconeogenesis (17). In addition, studies reveal that hepatic FoxO1 plays a role in T2D since liver-specific FoxO1 activation in transgenic mice increases glucose levels and impairs glucose tolerance (65). Furthermore, loss of hepatic FoxO1 function improves glycemia, mitochondrial function, and T2D symptoms in diabetic mice (17, 64, 66, 67).

Insulin decreases hepatic glucose production via the AKT/FoxO1 pathway and increases lipogenesis largely via activation of mechanistic target of rapamycin complex 1 (mTORC1), which activates the major lipid regulator sterol regulatory element binding protein 1c (SREBP1c) (68, 69). However, evidence indicates that FoxO1 also contributes to insulin’s control of lipid metabolism in the liver. For example, microsomal triglyceride transfer protein (MTP) and apolipoprotein apoC-III are proteins that associate with very low-density lipoprotein (VLDL) and regulate triglyceride transport. FoxO1 directly upregulates the transcription of both MTP and apoC-III (70, 71) (see **Figure 2**). Insulin stimulated AKT suppression of FoxO1 decreases MTP and apoC-III. Conversely, reduced activation of the PI3K/AKT pathway increases FoxO1 activity along with the production of MTP and apoC-III. Moreover, FoxO1 overactivation was found to cause VLDL accumulation in the liver, elevated plasma triglyceride levels, and an increased the risk of atherosclerosis (70, 71). However, conflicting results have been reported from studies using transgenic mice with overactive FoxO1 expression in the liver. For example, some studies show that elevated hepatic FoxO1 increases *de novo* lipogenesis and triglyceride levels, yet another study found that constitutively active hepatic FoxO1 decreases triglyceride and cholesterol levels (65, 72). Liver-specific knockout of FoxO1 and FoxO3 independently indicates that FoxO3 is the more substantial regulator of lipogenesis as FoxO3 deletion increased the expression of lipogenic enzymes. However, simultaneous deletion of FoxO1 and FoxO3 revealed a synergistic increase in the transcription of the lipogenic enzyme glucokinase (Gck), which lead to hepatic steatosis (17). Further research has demonstrated that FoxO1 associates with the corepressor SIN3 Transcription Regulator Family Member A (SIN3A) to downregulate Gck expression and decrease lipogenesis (73). These results suggest that in conditions in which FoxO1 activity is enhanced like during insulin resistance, FoxO1 should decrease lipid synthesis. Given that insulin resistance is often associated with hyperglycemia and hyperlipidemia, a pathogenic state termed “selective insulin resistance”, researchers have suggested that other regulators may play a more prominent role regulating lipid metabolism in T2D (74, 75). While FoxO1’s role in regulating gluconeogenesis is well established, more research is needed to elucidate FoxO1’s role in lipid metabolism.

Since FoxO1 contributes to hyperglycemia in T2D, research has focused on targeting drugs that inhibit hepatic FoxO1 as potential T2D therapeutics (e.g. 76). For example, the selective FoxO1 inhibitor AS1842856 reduces fasting plasma glucose levels in db/db diabetic mice. More recent studies have identified candidate treatments that mitigate T2D symptoms by enhancing AKT-mediated suppression of FoxO1. For example, the traditional Chinese herbal medicine, Simiao Wan, lowers blood glucose levels by enhancing AKT expression and decreasing FoxO1 expression (77). The dietary fiber Oat β-D-glucan has been shown to decrease blood glucose levels and enhance insulin sensitivity (78). Recently, Guo et al. (79) demonstrated that Oat β-D-glucan exerts its actions in part by lowing gluconeogenesis via increased PI3K/AKT and reduced FoxO1. Vitamin D deficiency has been associated with T2D and Vitamin D supplementation improves insulin sensitivity in insulin resistant individuals (80–83). Yuan et al. (84) demonstrated that Vitamin D3 directly upregulates hepatic SIRT1 transcription which activates AKT to inactivate FoxO1 to decrease gluconeogenesis and improve glycemia. Similarly, the nicotinamide metabolite N1-Methylnicotinamide (MNAM) lowers blood glucose and hepatic lipid accumulation by increasing SIRT1 and pAKT and decreasing FoxO1 activity (85). The bioactive lipid, prostaglandin E2 (PGE2) also reduces gluconeogenesis and ameliorates T2D symptoms by inhibiting FoxO1 activity (86). SW03329 is a small molecule that enhances PGE2 levels by inhibiting the PGE2-degrading enzyme 15-hydroxyprostaglandin dehydrogenase. SW033291 reduces gluconeogenesis, blood glucose, serum triglycerides and body weight in diabetic mice. These actions of SW033291on gluconeogenesis are mediated by increased AKT inactivation of FoxO1. The role of n3-polyunsaturated fatty acids (n3-PUFAs) on diabetes and nonalcoholic fatty liver disease (NAFLD) has received considerable attention, and a recent meta-review analysis concluded that n3-PUFAs act to prevent and treat T2D (87). Further research demonstrated that n3-PUFAs reduce hepatic FoxO1 expression to improve insulin sensitivity as well as glycemia and lipid profiles of diabetic mice (88). This study also showed that metformin, the popular first-line T2D drug, lowers FoxO1 expression in diabetic mice. Guo et al. (89) described the mechanism by which metformin acts on FoxO1 to reduce hepatic glucose production and blood glucose levels, which was not by regulating insulin signaling, but rather by affecting glucagon signaling. Glucagon is a hyperglycemic hormone that activates Protein Kinase A (PKA) to elevate gluconeogenesis and thus blood glucose levels. PKA acts as a FoxO1 activating kinase importing and retaining FoxO1 in the nucleus to increase gluconeogenesis (90). Metformin was found to lower blood glucose by inhibiting glucagon induced PKA activity causing a decrease in FoxO1 and gluconeogenesis. Altogether, these results advocate for the use of hepatic FoxO1 inhibition in T2D treatment.

## 4 FoxO1 and T2D – skeletal muscle

FoxO1 regulates muscle mass and glucose metabolism in skeletal muscle. For example, FoxO1 overexpression in transgenic mice results in reduced body weight and muscle mass as well as impaired glucose tolerance (91). FoxO1 upregulates the ubiquitin ligase atrogin-1 (also known as MAFbx) which enhances muscle atrophy (92). Furthermore, insulin-like growth factor (IGF) activation of the PI3K/AKT pathway suppresses FoxO1 and FoxO3 action on atrogin-1, which promotes muscle mass. Combined loss of FoxO1 and FoxO4 function in skeletal muscle increases the expression of atrogin-1 and causes muscle hypertrophy (93). Furthermore, evidence suggests that FoxO1 may also play a role in muscle atrophy during T2D. Hyperglycemia in T2D increases the levels of Advanced Glycation End Products (AGEs), which are toxic and exacerbate T2D symptoms (94). Du et al. (95) found that elevated AGE increases skeletal muscle atrophy by increasing OS and endoplasmic reticulum (ER) stress, which is caused by the accumulation of unfolded or misfolded proteins. ER stress activates PERK, which in turn activates FoxO1 to increase atrogin-1. SiRNA silencing of FoxO1 rescued the AGE effect on muscle atrophy signifying that AGE production in T2D may promote muscle wasting via FoxO1. Additionally, FoxO1 has been shown to decrease muscle mass by upregulating the ubiquitin ligase MuRF1 and the liposomal protease cathepsin L (96, 97). Skeletal muscle specific knockout studies further support a role for FoxOs in mediating muscle atrophy. For example, one study showed that the collective deletion of skeletal muscle FoxO1, FoxO3, and FoxO4 upregulates the SMART (Specific of Muscle Atrophy and Regulated by Transcription) group of ubiquitin ligases to protect against fasting-induced muscle wasting (98). Likewise, another study demonstrated that insulin and IGF act to increase muscle mass through their negative regulation of FoxO activity (99). This study showed that while insulin is the primary mediator of muscle protein synthesis, insulin and IGF work synergistically to increase muscle mass. Moreover, muscle specific knockout of both insulin and IGF (MIGIRKO) depletes muscle mass in a FoxO-dependent manner as simultaneous deletion of FoxO1, FoxO3, and FoxO4 in MIGIRKO mice reversed the loss of muscle mass. Notably, ageing patients with T2D have significantly reduced muscle mass, which is associated with increased mortality (100–102). A recent study showed that skeletal muscle specific knockout of AKT1 and AKT2 causes sarcopenia, the progressive reduction in muscle strength and mass, and insulin resistance both of which were largely reversed by additional knockout of FoxO1 (103). Future studies should determine whether targeting FoxO1 inactivation could treat T2D-induced muscle wasting.

The increase in blood glucose that characterizes T2D is largely caused by decreased insulin-stimulated glucose uptake into skeletal muscle via GLUT4 glucose transporters (104, 105). Evidence suggests that FoxO1 and FoxO3 play a role in insulin’s regulation of GLUT4. For example, Lundell et al. (106) found that decreased expression of either FoxO1 or FoxO3 in skeletal muscle reduces GLUT4 protein levels and glucose uptake. Furthermore, attenuation of FoxO3, and not FoxO1, decreased glycogen content indicating that FoxO3 is important for the control of glucose storage in skeletal muscle. Further studies suggest that both AKT and SIRT1 regulate FoxO1’s effects on muscle mass and GLUT4 (107). Elevated SIRT1 during cardiac or skeletal muscle hypertrophy has been associated with increased AKT levels and activity and decreased FoxO1 (61, 107–109). For example, Gombos et al. (61) found that experimentally induced skeletal muscle hypertrophy enhances SIRT1 and AKT levels, decreases FoxO1 levels, and increases GLUT4 levels. Altogether, studies show that increased skeletal muscle FoxO1 acts to elevate blood glucose by decreasing GLUT4 levels and thus reducing glucose uptake.

Reduced glucose uptake and increased lipid accumulation in skeletal muscle contributes to the shift from glucose oxidation to lipid oxidation observed in T2D (110–112). FoxO1 has been shown to mediate lipid metabolism in skeletal muscle. For example, a study using the C2C12 skeletal muscle cell line reported that FoxO1 upregulates lipoprotein lipase (LPL), which is an enzyme that hydrolyses triglycerides into fatty acids (113). In addition, FoxO1 alters the cellular localization of the fatty acid translocase CD36 to enhance fatty acid uptake (111). Specifically, overactivation of FoxO1, by impeding AKT-induced FoxO1 inactivation, increases CD36 localization in the plasma membrane along with fatty acid uptake into C2C12 skeletal muscle cells. Blocking AKT-mediated FoxO1 inactivation also promotes fatty acid oxidation. In addition to fatty acid oxidation, insulin activation of AKT regulates glucose oxidation in skeletal muscle. For example, insulin augments glucose oxidation by strongly suppressing pyruvate dehydrogenase kinase (PDK) 4, which is an enzymes that inhibits the conversion of pyruvate to acetyl-CoA thereby reducing glucose oxidation and ATP production (114). However, low insulin signaling conditions like fasting and diabetes increases skeletal muscle PDK4 (115). For example, rats induced with diabetes present with increased PDK activity in skeletal muscle as well as lower glucose oxidation, and insulin reverses these effects (116, 117). FoxO1 mediates insulin’s action on PDK4. For example, FoxO1 has been shown to directly upregulate PDK4 transcription in skeletal muscle (118). Furthermore, stimulation of acute insulin resistance in rats increases PDK4 expression, decreases the active phosphorylated form of AKT, and increases the inactive phosphorylated form of FoxO1 (112). Overall, these results suggest that increased FoxO1 activity in insulin resistance contributes to the decrease in skeletal muscle glucose uptake and utilization along with the increase in fatty acid uptake and oxidation.

Recent studies demonstrate that pharmacological inhibition of FoxO1 improves muscle mass (119). For example, treatment with the FoxO1 specific inhibitor AS1842856 partially reverses the loss of fast-twitch muscle mass caused by AKT1/2 deletion (103). Furthermore, FoxO1 inhibition also mitigates insulin resistance. For example, the traditional Chinese herbal treatment Geniposide, which is an iridoid glycoside found in Gardenia fruits, improves glucose utilization in skeletal muscle by downregulating FoxO1 and PDK4 expression (120). Similarly, the Chinese herbal formula Yunpi Heluo (YPHL) decoction was found to increase SIRT1 and decrease FoxO1 in skeletal muscle of diabetic Zucker rats. YPHL treatment in these rats also improves glycemia and insulin sensitivity, and lowers cholesterol, triglyceride levels, and body weight (121). Vitamin D is known to alleviate T2D symptoms (80–83). Studies indicate that Vitamin D3 improves T2D by targeting hepatic FoxO1 suppression (84). Recent work shows that lack of the vitamin D receptor in skeletal muscle increases FoxO1 activity and causes insulin resistance and glucose intolerance (83). Thus, the beneficial effects of Vitamin D on insulin sensitivity may also act via FoxO1 inactivation in skeletal muscle. Taken together, these results suggest that FoxO1 inhibition in skeletal muscle may help treat T2D.

## 5 FoxO1 and T2D – adipose tissue

FoxO1 is densely expressed in white adipose tissue (WAT), where it controls adipocyte differentiation from preadipocytes. FoxO1 decreases adipogenesis by acting as a transrepressor of peroxisome proliferator-activated receptor gamma (PPARγ) and as a transactivator of the cell cycle inhibitor p21 (122–124). However, siRNA silencing of FoxO1 as well as FoxO1 inhibition with the selective antagonist AS1842856 inhibits adipogenesis (125, 126). FoxO1’s complicated regulation of adipocyte differentiation is timing dependent and reflects its role in regulating the cell cycle (123). Despite this complexity, targeting FoxO1 in WAT for T2D treatment has proven effective. For example, reducing FoxO1 activity in insulin-impaired mice increases insulin sensitivity, and decreasing FoxO1 activity specifically in WAT enhances insulin sensitivity and glucose tolerance (122, 127, 128). Recent evidence reveals that regulation of adipocyte FoxO1 by 3′-phosphoinositide-dependent kinase 1 (PDK1) plays an important role in metabolic disease. PDK1 is a kinase involved in insulin signaling. Insulin binding its receptor activates PI3K, which phosphorylates AKT directly and indirectly through facilitating AKT activation by other kinases like PDK1. For example, PI3K increases phosphatidylinositol (3-5)-trisphosphate (PIP3) which recruits PDK1 to the plasma membrane and promotes the interaction between PDK1 and AKT (129, 130). PDK1 phosphorylates AKT specifically at Thr308 (**Figure 2**). FoxO1 activity is decreased with increased PDK1/AKT signaling. Adipocyte-specific PDK1 knockout (A-PDK1KO) mice manifest symptoms of metabolic disease including insulin resistance, glucose intolerance, and hepatic steatosis that resembles the phenotype generated by knocking out the insulin receptor in adipocytes (131). These results emphasize the importance of PDK1 in insulin’s metabolic actions in WAT. Furthermore, these A-PDK1KO metabolic changes were reversed in mice with a combined deletion of PDK1 and FoxO1 indicating that FoxO1 activity was required for the development of insulin resistance, glucose intolerance, and hepatic steatosis in mice lacking PDK1 (132). These data suggest that inhibiting FoxO1 activity during insulin resistance when PDK1 signaling is reduced may help to reverse T2D symptoms. Other agents have been shown to mitigate T2D symptoms via FoxO1 inhibition in WAT. For example, the nonsteroidal anti-inflammatory drug-activated gene-1 (NAG-1) exerts its anti-diabetic effects by reducing adipocyte FoxO1 (133). Similarly, serum- and glucocorticoid-inducible kinase 1 (SGK1) increases AKT phosphorylation of FoxO1 which increases glucose uptake in adipocytes, and thus improves insulin sensitivity (134). Photobiomodulation (PBMT) is a non-invasive therapy that uses light sources like low-level lasers to stimulate cell proliferation, alter signaling pathways, decrease inflammation, and relieve pain in conditions such as diabetes (135). PBMT may improve T2D by inhibiting FoxO1. For example, a recent study demonstrated that PBMT via abdominal laser treatment enhances AKT inactivation of FoxO1 which decreases free fatty acid production and release from adipocytes of obese and diabetic mice (136). Knockdown of prolyl 4-hydroxylase subunit alpha 3 (P4HA3) with siRNA increases pAKT and pFoxO1, which decreases the transcriptional capacity of FoxO1. Consequently, P4HA3 inhibits adipogenesis, decreases fasting blood glucose levels, improves insulin resistance, and decreases body weight in diabetic mice (137). Altogether, these studies demonstrate that FoxO1 inhibition in WAT may alleviate T2D symptoms.

FoxO1 also regulates lipolysis by directly upregulating the lipolytic enzyme adipose triglyceride lipase (ATGL) (123). Deacetylation of FoxO1 by SIRT1 augments this response (138). Adipose-specific knockdown of Sirtuin 6 increases the acetylated form of FoxO1 causing an decrease in FoxO1 activity and downregulation of ATGL, which results in adipocyte hypertrophy (without hyperplasia), and promotes insulin resistance in obese fed mice (139). Adipocyte hypertrophy has received attention as an important factor inciting metabolic dysfunction via increased inflammation which was also reported in this study (140). Additionally, WAT ATGL is the first identified biosynthetic enzyme of mammalian fatty acid esters of hydroxy fatty acids (FAHFAs) some of which have anti-diabetic actions further highlighting FoxO1 and ATGL as therapeutic targets for T2D treatment (141). More research is needed to determine whether FoxO1’s regulation of lipolysis and FAHFAs via AGTL could prove effective in T2D treatment.

Brown adipose tissue (BAT) is a target for T2D treatment because of its ability to increase basal metabolic rate and decrease body weight. FoxO1 impedes BAT function by downregulating PPARγ coactivator (PGC)-1α and uncoupling protein 1 (UCP1) (128). FoxO1 also blocks the conversion of WAT to BAT by upregulating transcription factor EB (TFEB) (142) (**Figure 3**). The role of FoxO1 in regulating WAT and BAT depends on timing and insulin signaling. For example, Homan et al. (143) showed that knocking out FoxO1, FoxO3, and FoxO4 in adipocytes during early development of mice lacking the insulin and IGF receptors increases BAT mass and function, partially increases WAT, intensifies hyperinsulinemia, and improves hepatic insulin sensitivity. Future research should characterize the specific roles of each FoxO and determine whether these changes are dependent on developmental timing. Pharmacological intervention with the FoxO1 inhibitor AS1842856 decreases TFEB and increases UCP1 to increase brown adipocyte differentiation and function. Furthermore, Zhuang et al. (137) demonstrated that metformin inhibits autophagy and promotes BAT differentiation in adipose-derived stem cells by FoxO1 inactivation. Taken together, evidence supports FoxO1 inhibition in BAT as an attractive target for T2D therapeutics.

## 6 FoxO1 and T2D – pancreatic β-cells

Pancreatic β-cells produce and secrete insulin. These cells are particularly sensitive to the development of insulin resistance. Indeed, early insulin resistance induces a compensatory response in which insulin production and secretion is enhanced in part due to β-cell hyperplasia. Enhanced insulin signaling during compensatory hyperinsulinemia increases the activity of pancreatic duodenal homeobox 1 (PDX1) which is a transcription factor that augments β-cells proliferation (144). Kulkarni et al. (144) showed that PDX1 deficiency impairs insulin’s compensatory response on β-cell mass. Further work demonstrated that FoxO1 links insulin and PDX1 action on β-cells proliferation (145). FoxO1 suppresses the transcriptional activity of PDX1 (146, 147). Mechanistically, compensatory hyperinsulinemia increases pancreatic PI3K/AKT signaling, which decreases FoxO1 activity and causes an increase in PDX1 activity and β-cell mass (145). However, if this response fails to compensate and insulin resistance persists, β-cell proliferation will decrease along with insulin secretion and T2D will develop. In this state of advanced insulin resistance, the decrease in insulin lowers PI3K/AKT signaling and increases FoxO1 activity which impairs PDX1-mediated β-cell growth further reducing insulin secretion and contributing to T2D pathogenesis.

In addition, microRNA (miRNA) studies have signified a role for FoxO1 in T2D. For example, FoxO1 upregulates miR-802, which is elevated in diabetic mouse pancreatic islets and contributes to β-cell dysfunction by decreasing insulin secretion (148). Li et al. (149) showed that miR-233 is upregulated in pancreatic cells of diabetic mice and humans and improves β-cell proliferation and function via FoxO1 suppression. Similarly, an irisin-induced increase in miR-133a-3p downregulates FoxO1 to protect β-cells from pyroptosis (150). Pharmacological inhibition of β-cell FoxO1 also ameliorates T2D symptoms. For example, the anti-allergic drug and FoxO1 inhibitor tranilast improves glucose tolerance in diabetic mice and reverses palmitic acid (PA)-induced reduction in β-cell insulin secretion (151). Similarly, glucagon-like peptide-1 receptor agonists (GLP-1RA) mimic incretin hormones, which increase insulin secretion and β-cell proliferation through PI3K/AKT-mediated nuclear exclusion of FoxO1 (152, 153). For example, the GLP-1RAs liraglutide and EXf inactivate FoxO1 causing increased β-cell proliferation in a PI3K-dependent manner (154, 155). Additionally, the isoflavone puerarin increases GLP-1R activation of AKT, decreases FoxO1 activity, and improves glucose tolerance (156, 157). Vitamin D also improves insulin secretion via reduced FoxO1 protein levels which reduces β-cell ferroptosis (158). Furthermore, Zhang et al. (159) reversed the negative effects of β-cell senescence using the *Saccharina japonica* derivative fuco-manno-glucuronogalactan (SFGG) in insulin secreting MIN6 cells. SFGG anti-senescence effects are regulated by pAKT-mediated FoxO1 suppression suggesting that FoxO1 contributes to the T2D pathogenesis by accelerating β-cell ageing. FoxO1 deficiency may also contribute to β-cell senescence and T2D as knockout of the proliferation mediator SMAD7 reduced FoxO1 which enhanced β-cell ageing and caused diabetes suggesting that there may be an optimal range of FoxO1 activity (160).

FoxO1 may help to prevent T2D under conditions like OS and inflammation which are associated with T2D pathogenesis. For example, FoxO1 protects β-cells from OS by upregulating antioxidants and by interfering with Carbohydrate Response Element-binding Protein (Chrebp) to combat DNA damage and apoptosis (2, 5, 19, 161, 162). In contrast to AKT, phosphorylation of FoxO1 by inflammatory pathways like c-Jun N-terminal kinase (JNK) augments nuclear entry of FoxO1 to increase insulin transcription via upregulation of NeuroD and MafA transcription factors (163, 164). Moreover, studies indicates that as insulin resistance and OS progresses, FoxO1 acts to combat these conditions but over time its protein levels decline causing β-cell dedifferentiation and marking a molecular switch to T2D pathology (165, 166). Furthermore, the GLP-1RA exenatide decreases inflammation and boosts β-cell function by improving the balance between CD4+ T-helper 17 (Th17) cells and regulatory T-cells (Tregs) (167). The FoxO1 inhibitor AS1842856 reversed these effects indicating that FoxO1 activity may be important for T17/Treg balance and reduced inflammation. FoxO1 also safeguards β-cells during hypoxia by regulating autophagy (168). For example, liraglutide protects INS-1 cells from PA-induced injury by enhancing autophagy and cell survival through elevated FoxO1 activity (169). Similarly, Liang et al. (170) demonstrated that FoxO1 stimulates autophagy and enhances survival in cells under T2D-induced hypoxia.

FoxO1 knockout studies have examined its dual role in T2D. For example, FoxO1 deletion in mouse β-cells causes mitochondrial dysfunction, cell dedifferentiation, and hyperglycemia (171, 172). Kobayashi et al. (173) showed that knocking out FoxO1 in the pancreas increases β-cell mass, but this was not the case when FoxO1 was deleted specifically from β-cells. Furthermore, glucose intolerance in db/db diabetic mice was worsened when FoxO1 was knocked out in the pancreas as well as when FoxO1 was knocked out explicitly in β-cells. These data show that while FoxO1 inhibits β-cell proliferation, it can also improve glucose tolerance. Therefore, activating FoxO1 could prove effective. For example, L-Methionine (L-Met) regulates FoxO1 by altering the bivalent domain histone methylation marks H3K27me3 and H3K4me3 to increase FoxO1-mediated upregulation of the insulin transcription factor MafA and mitigate type 1 diabetes mellitus in rats (174). Given that Obex^®^, a supplement containing L-Met, caused weight loss and improved insulin homeostasis in overweight and obese participants of a double−blind, randomized, controlled phase III clinical trial, future studies should explore how altering bivalent domain controls FoxO1 regulation of β-cells during T2D (175).

## 7 FoxO1 and T2D – brain

FoxO1 mediates the hypothalamic control of metabolism by integrating signals from peripheral regulators like ghrelin, insulin, and leptin (176). Ghrelin-activated FoxO1 in the hypothalamic arcuate nucleus (ARC) induces hyperphagia by upregulating the orexigenic peptide Agouti-related protein and downregulating proopiomelanocortin (POMC) from which the anorexigenic peptide alpha melanocyte stimulating hormone (α-MSH) is derived (177). FoxO1 also decreases the POMC processing enzyme Carboxypeptidase E (Cpe) further reducing α-MSH (178). FoxO1 ablation in POMC neurons increases CPE and α-MSH resulting in reduced food intake and protection from diet-induced obesity (DIO) weight gain. In contrast, FoxO1 overexpression in hypothalamus and pancreas increases food intake and impairs glucose tolerance and insulin secretion (179). Leptin and insulin reverse FoxO1 action by enhancing AKT inactivation of FoxO1 (33, 180). However, leptin and insulin signaling is compromised in obesity and T2D in the ARC (181). Therefore, pharmacologically inactivating hypothalamic FoxO1 could treat these conditions. Indeed, central inhibition of FoxO1 directly or via SIRT1 inhibition reduces body weight and improves insulin sensitivity in DIO rodents (182, 183). Furthermore, actions of the orexigenic melanin-concentrating hormone (MCH) in the ARC that promote hyperphagia, adipocyte lipid storage, and glucose intolerance are dependent on SIRT1 and FoxO1 activation (184). Overall, evidence suggests that FoxO1 inhibition in the hypothalamus has the potential to treat obesity induced T2D.

Because of FoxO1’s role in regulating brain metabolism, it may link metabolic dysfunction and neurodegenerative disease (185). For example, neurodegenerative diseases like Alzheimer’s disease (AD), Huntington’s disease (HD), and Parkinson’s disease (PD) are worsened by T2D (186–188), and FoxO1 expression is elevated in each of these conditions (189–193). Furthermore, increased FoxO1 has been shown to reduce pathological AD features such as tau hyperphosphorylation and amyloid beta (Aβ) plaques (190, 194–196). A recent study screened FoxO1 activators as potential therapeutics and demonstrated that one, Compound D, decreases Aβ1-40 and Aβ1-42 in SH-SY5Y cells (197). Additionally, exercise, an AD intervention, increases circulating FoxO1 in African American men with mild cognitive impairment (198), and upregulates FoxO1 to improve AD symptoms in early-onset AD mice (199). Interestingly, the ani-aging protein klotho improves AD symptoms by either activating (200, 201) or inactivating FoxO1 (202). Conflicting results of FoxO1 have also been reported in HD studies. For example, Li et al. (203) showed that AKT-mediated FoxO1 inactivation increases pathogenic mutant Huntington (mHtt) protein aggregates, yet another study showed that increasing FoxO1-dependent expression of autophagy, mitochondrial, and antioxidants genes improves HD symptoms including increased neuronal survival and enhanced motor control (204). Most studies of PD demonstrate that increased FoxO1 contributes to its pathology via increased inflammation and apoptosis (205, 206). Evidence also suggests that FoxO1 mediates the depressive symptoms of PD (207). More research is needed to better understand FoxO1’s role in the pathology of neurodegenerative diseases.

## 8 Conclusion and prospective

Insulin resistance in T2D increases FoxO1 activation in metabolic tissues including liver, skeletal muscle, adipose tissue, pancreas, and brain, which underscores FoxO1’s potential clinical relevance. However, recent evidence in these tissues and others like heart and kidney (208, 209) highlights controversy and indicates that inactivating FoxO1 could either mitigate or exacerbate T2D pathology (2–5). Knowing a patient’s history and risk for certain diseases may be critical. For example, FoxO1 appears to play opposing roles in AD and PD pathology at least under certain conditions. Furthermore, FoxO1 can act as a tumor suppressor and thus activating FoxO1 may help treat certain cancers, yet FoxO1 activation worsens stroke pathology (210, 211). Therefore, targeting the optimal tissue for activation or inhibition of FoxO1 and developing strategies for tissue-specific delivery of FoxO1 therapeutics could effectively treat T2D and minimize adverse side effects. Likewise, this approach could extend to targeting FoxO1 for treatment of other diseases.

## Author contributions

NC: Conceptualization, Supervision, Writing – original draft, Writing – review & editing. NT: Writing – original draft, Conceptualization, Writing – review & editing.
